# T-Cell Subpopulations and Differentiation Bias in Diabetic and Non-Diabetic Patients with Chronic Kidney Disease

**DOI:** 10.3390/biomedicines13010003

**Published:** 2024-12-24

**Authors:** Ana Cecilia Granda Alacote, Gabriela Goyoneche Linares, María Gracia Castañeda Torrico, Daysi Zulema Diaz-Obregón, Michael Bryant Castro Núñez, Alexis Germán Murillo Carrasco, Cesar Liendo Liendo, Katherine Susan Rufasto Goche, Víctor Arrunátegui Correa, Joel de León Delgado

**Affiliations:** 1 Faculty of Natural Sciences and Mathematics, Universidad Nacional Federico Villarreal, Lima 15001, Peru; grandaalacote.ana@gmail.com (A.G.A.); gabriela.goyoneche.l@gmail.com (G.G.L.); 2 ONG Innovation and Science for the Care and Support of Society–INNOVACARE, Lima 15036, Peru; maria_castaneda7@usmp.pe; 3 Faculty of Human Medicine, University of San Martín de Porres, Lima 15011, Peru; 4 Health Technology Assessment and Research Institute-EsSalud, Lima 15072, Peru; daysiz.diaz.o@gmail.com; 5 Postgraduate School, Universidad Nacional Federico Villarreal, Lima 15001, Peru; krufasto@unfv.edu.pe; 6 Faculty of Medicine, Universidad Nacional Federico Villarreal, Lima 15001, Peru; dr.michael.castro@gmail.com; 7 Postgraduate School, Universidad Nacional Mayor de San Marcos, Lima 15011, Peru; 8 Immunology and Cancer Research Group-IMMUCA, Lima 15001, Peru; agmurilloc@usp.br; 9 Nephrology Center-CENESA, Lima 15001, Peru; cesarliendo2@gmail.com; 10 Faculty of Dentistry, Universidad Nacional Federico Villarreal, Lima 15001, Peru; 11 Center of Virology Research, Faculty of Human Medicine, University of San Martín de Porres, Lima 15011, Peru

**Keywords:** chronic kidney disease, type 2 diabetes, transcription factors, T cells

## Abstract

Background: Chronic kidney disease (CKD) patients often experience dysregulated inflammation, particularly when compounded by comorbidities such as type 2 diabetes (T2D). Objective: The aim of this study was to determine whether T2D influences the profile of memory T lymphocytes, regulatory T cells (Tregs), and the gene expression of transcription factors such as *T-bet (Tbx21)*, *GATA3*, *RORyT (RORC)*, and *FOXP3* in CKD patients. Methods: Twenty-two CKD patients undergoing hemodialysis were selected for the study. Flow cytometry was used to identify naïve T cells, Tregs (CD4+CD25+CD127-), central memory T lymphocytes (CCR7+CD45RA-), effector memory T lymphocytes (CCR7-CD45RA-), and TEMRA cells (CCR7-CD45RA+). The expression of helper T cell differentiation regulatory genes was assessed using real-time RT-PCR. Results: Both helper and cytotoxic effector memory T cell populations were found to be higher than naïve lymphocytes in CKD patients, regardless of T2D status. However, Tregs were significantly more frequent in diabetic CKD patients (5.1 ± 2.6%) compared to non-diabetic patients (2.8 ± 3.1%). In terms of transcription factor expression, a significant correlation was observed between T-bet and *FOXP3* in diabetic patients, and between RORyT and FOXP3 in non-diabetic patients. Conclusions: While T2D does not notably alter the distribution of memory T cells in CKD patients, it significantly impacts the frequency of Tregs and their correlation with pro-inflammatory transcription factors like *T-bet (Tbx21)* and *RORyT*.

## 1. Introduction

Over one million deaths occur worldwide each year due to chronic kidney disease (CKD) [1]. The prevalence of CKD is rising and affects approximately 15% of the elderly population [2]. CKD patients often present with comorbidities, particularly type 2 diabetes (T2D), which contribute to additional complications such as retinopathy, cardiovascular diseases, and lower limb amputation [3]. Globally, T2D affects 7.2% to 11.3% of the population [4]. Beyond health implications, T2D-related complications account for more than 80% of CKD treatment costs [5], making this a significant public health challenge [6].

The role of the immune system in disease progression has garnered considerable attention, especially due to the success of immunotherapy [7]. Differentiation of helper T cells (Th) into Th1, Th2, Th17, and regulatory T cells (Tregs) plays a crucial role in the progression of various diseases, including cancer, metabolic disorders, and autoimmune conditions [8]. These T cells are regulated by master transcription factors such as *T-bet*, *GATA3*, *RORγT*, and *FOXP3*, respectively [9]. T-bet (encoded by the *Tbx21* gene) and *GATA3* are polarizing transcription factors that induce cross-regulation [10]. Th1 cells are pivotal in defending against intracellular pathogens and regulating IFNγ production [11], while Th2 cells are crucial for defending against helminth infections and allergens [12]. RORγT promotes the differentiation of thymocytes into Th17 cells, which secrete pro-inflammatory cytokines [13]. FOXP3, a marker of anti-inflammatory Treg cells, plays a key role in the immune response contraction and is relevant in autoimmunity control, cancer progression, and transplant tolerance [14].

Characterizing Th subpopulations and cytotoxic CD8+ T cells can provide insights into the immune system’s role in CKD. Previous studies have shown a significant reduction in naïve and central memory T cells (TCM) in patients with terminal CKD, while effector memory T cells (TEM) remained unchanged [15]. Additionally, both healthy aging and CKD lead to a similar reduction in naïve versus memory T cells, but Treg percentages are lower in CKD patients [16]. In diabetic nephropathy (DN), an increased frequency of Th1 and Th17 cells has been observed, along with a reduction in Treg and Th2 cells, suggesting that the Th17/Treg ratio may serve as an indicator of the immune microenvironment in DN [17,18]. The accumulation of senescent cells in T2D patients has been associated with an increased susceptibility to infections, impaired wound healing, and disrupted leukocyte migration [19]. Rattik et al. (2018) found that T2D patients with cardiovascular disease had an increased number of TEM cells producing IFN-γ [20].

The early stages of kidney function loss are often asymptomatic, making it essential to identify diagnostic and prognostic biomarkers for early detection and to assess the risk for dialysis or kidney transplant [21,22,23]. While previous research has confirmed the role of T cell differentiation bias in inflammatory diseases like CKD [15,17], this study offers a detailed analysis of T cell subtypes and Th differentiation bias in the context of T2D, a key comorbidity in Peruvian CKD patients.

## 2. Materials and Methods

### 2.1. Study Design

This observational, proof-of-concept, cross-sectional, and descriptive study evaluated CKD patients, with or without T2D, undergoing hemodialysis (HD) at a Peruvian nephrology center (CENESA). Patients with hematological, oncological, or autoimmune diseases, as well as those with a diagnosis or suspicion of type 1 diabetes or pregnancy, were excluded. Eleven CKD patients with T2D and eleven without T2D were included. Clinical and demographic data, such as gender, age, etiology, uremia, HD duration, body mass index, vascular access type, comorbidities, and blood counts, were obtained from the patients’ medical histories and anonymized. The study was approved by the Ethics Committee of the Faculty of Human Medicine at the University of San Martin de Porres (approval number: 917 2019-CIEI-FMII-USMP). All participants provided informed consent.

### 2.2. Isolation of Peripheral Blood Mononuclear Cells (PBMCs)

Blood samples collected in EDTA tubes were diluted 1:2 in phosphate-buffered saline (PBS). Viable PBMCs were isolated using Ficoll’s density gradient (Histopaque^®^-1077, Sigma-Aldrich, St. Louis and Burlington, MA, USA) by centrifugation. The cells were washed twice with PBS and resuspended in 200 µL of PBS.

### 2.3. Flow Cytometry

First, 1–2 × 10^6^ PBMCs were resuspended in 200 µL of 1X FACS solution (catalog number 349202, BD FACSTM). To detect memory T cells, PBMCs were stained with FITC-conjugated anti-CCR7 (catalog number 353215, Biolegend^®^, San Diego, CA, USA), PE-conjugated anti-CD45RA (catalog number 304107, Biolegend^®^, San Diego, CA, USA), PercP Cy5.5-conjugated anti-CD8 (catalog number 344709, Biolegend^®^, San Diego, CA, USA), PECy7-conjugated anti-CD3 (catalog number 317334, Biolegend^®^, San Diego, CA, USA), and APC-conjugated anti-CD4 (catalog number 357407, Biolegend^®^, San Diego, CA, USA). For Tregs, PBMCs were stained with FITC-conjugated anti-CD4 (catalog number 317407, Biolegend^®^, San Diego, CA, USA), PE-conjugated anti-CD25 (catalog number 302605, Biolegend^®^, San Diego, CA, USA), and APC-conjugated anti-CD127 (catalog number 351316, Biolegend^®^, San Diego, CA, USA). Gating and compensation were performed using irrelevant isotype control antibodies conjugated to FITC, PE, PercP-Cy5.5, PECy7, and APC. Cells were incubated at 4 °C for 20 min, washed twice with FACS solution, and resuspended in 0.3 mL of the solution. Flow cytometry was conducted using a BD FACSLyric™ Clinical Flow Cytometry System. The absolute number of T cell subpopulations was extrapolated from flow cytometry percentages and hematological analysis.

### 2.4. RNA Isolation and cDNA Synthesis

PBMCs were homogenized in Trizol reagent (Invitrogen™, Waltham, MA, USA) following the manufacturer’s instructions (ThermoScientific™, Waltham, MA, USA). After chloroform extraction, RNA was precipitated with isopropanol, washed with ethanol, and the pellet was resuspended in ultrapure water. RNA concentration was determined using a Qubit^®^ 2.0 fluorometer. cDNA synthesis was performed using the Maxima First Strand cDNA Synthesis Kit (ThermoScientific™, Waltham, MA, USA).

### 2.5. Evaluation of Transcription Factors Expression

The expression of *GATA3*, *FOXP3*, *RORγT (RORC*), and *T-bet (Tbx21)* genes was initially assessed in 29 immune cell subtypes using RNA-seq data from Monaco et al. [24] (Table 1). Quantitative real-time PCR (qPCR) was performed using SYBRGreen^TM^ reagents (Maxima SYBR Green/ROX qPCR Master Mix, ThermoScientific™, Waltham, MA, USA) and a Light Cycler 480 thermal cycler. Gene expression levels were normalized to ACTB as the endogenous control, applying the −ΔΔCt method [25]. Data analysis was performed using PE Applied Biosystems software and the dXpress web tool [26].

### 2.6. Statistical Analysis

Statistical analyses were performed using STATA version 12 and R version 4.4.0 software. Significant differences (*p* < 0.05) between diabetic and non-diabetic CKD patients undergoing hemodialysis were assessed. Depending on the normality of the data distribution, either the T-test or the Mann–Whitney U test was applied to compare mean values between groups. For categorical variables, the Chi-square or Fisher Exact test was used. Spearman’s test was employed to evaluate correlations between numerical variables. Given the limited data on gene expression in CKD patients with T2D, we hypothesized a large effect size (Cohen’s d-value = 0.95) as a proof of concept. Using a significance level of 0.1, the Statistics Kingdom tool [28] was used to calculate an estimated statistical power of 70% for this study.

## 3. Results

### 3.1. Study Cohort

Table 2 provides a detailed overview of the demographic and etiological characteristics of the CKD patients included in this study. Arterial hypertension and hepatitis C infection were the most common comorbidities observed. A statistically significant difference was found in the duration of uremia, with non-diabetic patients undergoing longer hemodialysis treatment. Regarding the etiology of End-Stage Renal Disease (ESRD), diabetic nephropathy was the predominant cause among diabetic patients, while glomerulonephritis and hypertensive nephropathy were more common among non-diabetic patients.

### 3.2. Comparative Analysis of CD4+ and CD8+ Memory T Lymphocytes in Diabetic and Non-Diabetic CKD Patients

**Figure 1:**
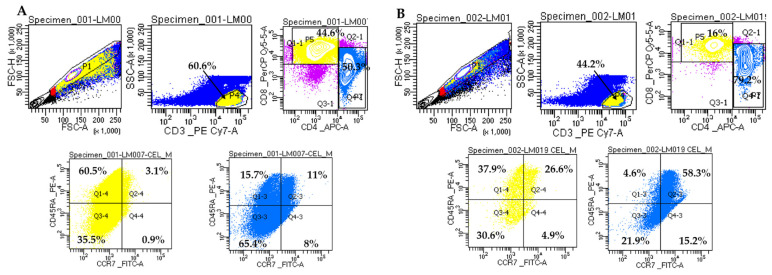
Representative analysis of memory T lymphocytes on two CKD patients. (**A**). Sample LM007 (non-diabetic patient). (**B**). Sample LM019 (diabetic patient). Gating strategy for the memory T cell panel: CD3+ cells were gated in P4 from FSC/SSC lymphocyte distribution; CD4+ and CD8+ T cells were gated in P7 and P5, respectively. CD8+ T lymphocytes were grouped as follows: naïve lymphocytes (CCR7+CD45RA+) in Q2-4, TCM central memory lymphocytes (CCR7+CD45RA-) in Q4-4, memory lymphocytes TEM effector lymphocytes (CCR7-CD45RA-) in Q3-4 and TEMRA effector lymphocytes (CCR7-CD45RA+) in Q1-4. CD4+ T lymphocytes were grouped as follows: naïve lymphocytes (CCR7+CD45RA+) in Q2-3, TCM central memory lymphocytes (CCR7+CD45RA-) in Q4-3, memory lymphocytes TEM effector lymphocytes (CCR7-CD45RA-) in Q3-3, and TEMRA effector lymphocytes (CCR7-CD45RA+) in Q1-3. Doublets were excluded by plotting FSC-H vs. FSC-A. Cells were analyzed on BD FACSLyric™ Clinical Flow Cytometry System.

No statistical difference was observed in the memory T cells mean number between diabetic and non-diabetic CKD patients (Table 3). Figure 1 shows the flow cytometric analysis of the memory T lymphocyte populations on a representative patient of each group.

### 3.3. Comparison of Treg Cells in Diabetic and Non-Diabetic CKD Patients

The absolute cell counts and the percentage of Treg cells were determined based on CD3+ lymphocytes gate in diabetic and non-diabetic CKD patients. As shown in Table 4, Treg CD4+CD25+CD127- and CD4+CD25+ cell counts show no significant differences between diabetic and non-diabetic patients. However, a statistical difference was observed in the percentages of Treg cells (*p* ˂ 0.05).

**Figure 2:**
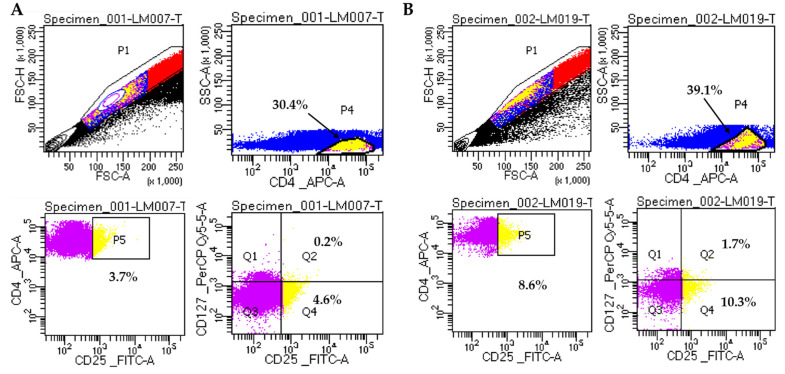
Representative analysis of Treg cells on two CKD patients. (**A**). Sample LM007 (non-diabetic patient). (**B**). Sample LM019 (diabetic patient). Gating strategy for Treg cells: CD4+CD25+ cells (P5) were selected from CD4+ cells (P4); CD4+CD25+CD127- Treg cells are shown in Q4. Doublets were excluded by plotting FSC-H vs FSC-A. Cells were analyzed on BD FACSLyric™ Clinical Flow Cytometry System.

Figure 2 shows the analysis of Treg lymphocytes corresponding to a representative patient of each group.

### 3.4. Normalized Expression of T-Bet (Tbx21), GATA3, RORyT (RORC), and FOXP3 Genes in PBMC Subpopulations

Considering that proportions of CD4+ T subpopulations could be imbalanced between diabetic and non-diabetic CKD patients, we explored the so-called master regulator genes to characterize CD4+ T cells differentiation bias on this patient cohort. Initially, we settled basal levels of the four genes in PBMCs subtypes based on the study of Monaco et al. [24].

**Figure 3:**
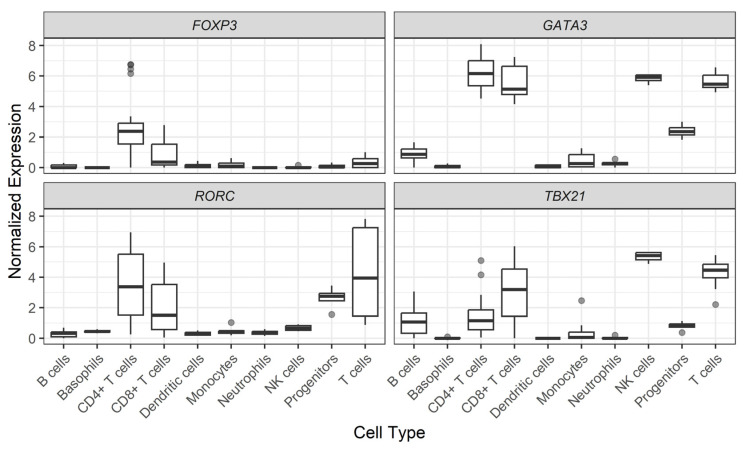
Normalized expression of gene levels across PBMC subtypes. Data are shown in log_2_ of Transcripts per Million (TPM) + 1. Cell subtypes were analyzed as originally described in the Monaco et al. study [25].

Figure 3 shows *FOXP3*, *GATA3*, *RORC*, and *Tbx21* gene expression levels among 10 representative cell subtypes found in human PBMCs. As expected, helper T cells differentially express *FOXP3*, *GATA3*, *RORC*, and *Tbx21* genes. However, Natural Killer (NK), progenitor, and B cells express certain levels of these genes. In particular, a significant *GATA3* and *Tbx21* expression was found in NK cells. For more cell subtypes, please refer to Figure S1.

### 3.5. Expression of Tbx21, GATA3, RORC, and FOXP3 Genes in Diabetic and Non-Diabetic CKD Patients

No significant difference was observed in diabetic compared to non-diabetic CKD patients regarding the expression of the transcription factors that control CD4+ T lymphocytes differentiation bias (Table 5). However, patients with CKD express more Tbx21 (median level −7.52 ± 1.93) than *GATA3* (median level −12.3 ± 2.08), in opposition to the behavior of these genes in healthy individuals (Figure 3).

**Figure 4:**
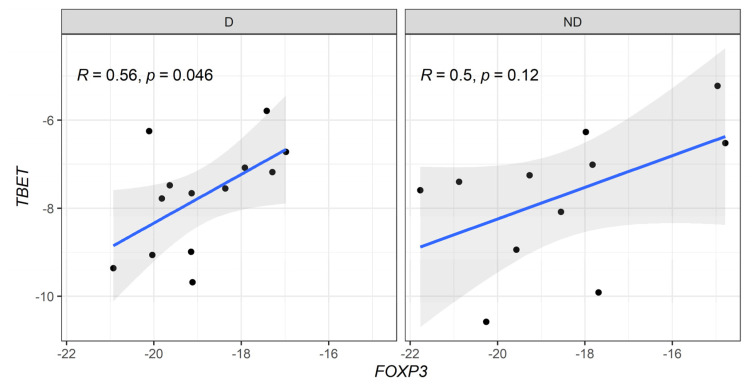
Correlative expression (−ΔCt) of *FOXP3* and *T-bet* (*Tbx21*) genes on diabetic (D) and non-diabetic (ND) CKD patients. Correlation values were calculated with the Spearman test.

**Figure 5:**
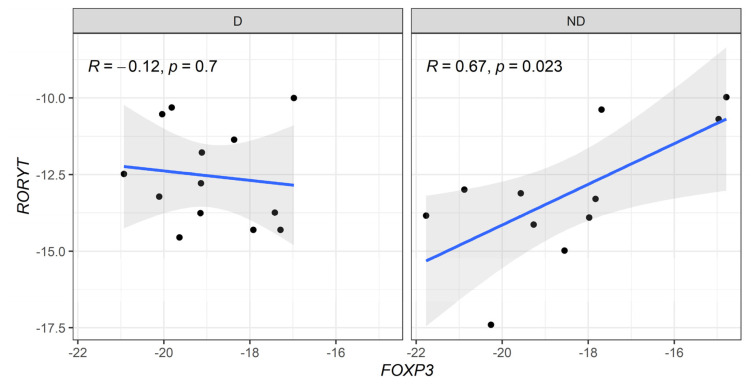
Correlative expression (−ΔCt) of *FOXP3* and *RORyT* (*RORC*) genes on diabetic (D) and non-diabetic (ND) CKD patients. Correlation values were calculated with the Spearman test.

When correlation profiles were evaluated between anti-inflammatory (*FOXP3*) and pro-inflammatory (*Tbx21* and *RORC*) genes, a moderate positive correlation was detected in *FOXP3* vs. *Tbx21* in diabetic CKD patients (Figure 4). In addition, a strong positive correlation between the expression levels of *FOXP3* vs. *RORC* genes was observed in non-diabetic patients (Figure 5).

## 4. Discussion

T cell differentiation profiles that reflect inflammatory conditions are crucial for tracking chronic diseases such as diabetes and kidney failure. Patients with T2D exhibit higher percentages of TEM and TCM cells compared to non-diabetic patients, while their frequency of naïve T cells is lower [20]. Aging and terminal CKD are associated with premature declines in memory cell function [16]. CKD patients show a significant reduction in naïve and TCM cells due to apoptosis [15]. Naïve T cells are more vulnerable to apoptosis in a uremic environment, contributing to their decline [29]. Moreover, differences in T cell frequencies influenced by dialysis modality and age have been noted; CKD patients on dialysis have a lower frequency of CD8+ TCM and CD4+ TCM cells compared to those on peritoneal dialysis [30].

Dialysis therapy does not resolve the immunological dysregulation caused by uremia [31]. The retention of uremic molecules and cytokines in terminal-stage CKD patients exacerbates oxidative stress and inflammation, leading to T cell deterioration and premature immune aging. Similarly, no reversal of T cell immunological aging has been observed in renal transplant patients [32]. Monitoring T cells in CKD patients is essential, as peripheral naïve T cells can predict post-transplant survival [33]. Furthermore, kidney transplant recipients with high frequencies of CD8+ TEMRA cells have double the risk of renal dysfunction [34].

We investigated whether T2D influences the abundance of T cell subtypes (TEM, TEMRA, TCM, naïve T) in CKD patients. While we hypothesized that T2D as a comorbidity might alter T cell profiles, no significant differences in T cell subset numbers were detected. Additionally, the frequency of naïve and TCM CD8+ T cells was lower than TEM and TEMRA CD8+ T cells, regardless of T2D status. In terms of Th cells, TEMRA and TCM numbers were lower than naïve and TEM cells. Imbalanced CKD and hemodialysis duration between experimental groups may explain our results, as longer dialysis exposure may subject non-diabetic patients to subclinical infections, increasing memory T cells.

According to Freitas et al., the average number of Treg cells in adult CKD patients is lower than in healthy controls [16]. Although the number of CD4+CD25+CD127- Treg cells in diabetic CKD patients was higher than in non-diabetic CKD patients, no significant difference was found (*p* > 0.05). However, a statistically significant increase in the percentage of Tregs was observed in diabetic CKD patients. Similar findings were reported in T2D patients with cardiovascular disease compared to those without [20]. Another study on diabetic nephropathy patients showed that Treg percentages were higher than in healthy controls [18]. This remains controversial since Treg counts were not linked to diabetes duration and increased immune activation, inflammation, or T2D progression [17]. It is worth noting that CD25, also an activation marker on conventional CD4+ T cells, may have altered expression in CKD and T2D patients. Thus, detecting FOXP3 in CD4+CD25+CD127- Treg cells would be relevant.

The balance between Th1, Th2, and Th17 differentiation is vital for maintaining normal immune function. An imbalance can contribute to cancer, autoimmunity, allergies, metabolic infections, and organ transplant rejection. Studies on Th cell profiles have generally concluded that T2D patients experience increases in Th1 and Th17 cells, which correlate positively with creatinine levels and diabetic nephropathy progression [17]. An inverse correlation between disease progression and Treg cell expression has also been documented [35].

When we compared the genetic expression of master regulatory transcription factors FOXP3, GATA3, RORC, and T-bet (Tbx21), no significant differences were found between CKD patients with and without T2D. However, a significant positive correlation between T-bet and FOXP3 was observed in diabetic patients, suggesting that Th1 differentiation is counteracted by Treg cells. This dynamic has been recognized as crucial in the pathogenesis of glomerulonephritis [36]. Indeed, an increased Th17/Treg ratio has been associated with renal function deterioration and CKD progression, driven by inflammation-induced tissue damage [37]. In non-diabetic CKD patients, we found elevated RORC expression correlating with FOXP3, suggesting exposure to infectious agents due to longer hemodialysis durations compared to diabetic patients.

In diabetic CKD patients, a notable but statistically insignificant imbalance (chi-square; *p* = 0.1932) was detected between male and female patients. Gender does not appear to influence immune cell differences in CKD patients undergoing HD. The decline in renal function in CKD leads to the accumulation of uremic toxins, affecting the hypothalamic-pituitary-gonadal axis, which reduces gonadotropin production and, consequently, hormone levels. This process can result in hypogonadism in men and premature menopause in women [38]. Al-Khafaji (2020) found that gender did not significantly influence immune cell differences in these patients [39].

A fourth-level care institution was selected for this study, as CKD patients receive optimal supportive care during HD treatment. Additionally, patients undergo frequent hematological evaluations, and detailed clinical records are accessible. While the number of available patients is limited, conclusions are based on robust experimental techniques designed for single-cell evaluation. These techniques revealed a decrease in naïve T cells, an increase in TEM cells, higher expression of T-bet and RORC, and reduced expression of *FOXP3* in CKD patients. The borderline *p*-values between diabetic and non-diabetic patients may be partially explained by the small sample size. Therefore, ongoing studies aim to validate phenotypic and genetic T cell subtype characteristics in a larger cohort of diabetic and non-diabetic CKD patients.

## Figures and Tables

**Table 1:** Oligonucleotide sequences.

Gene	Size (bp)	Sequence
*T-bet (TBX21)*	107	CGG CTG CAT ATC GTT GAG GT
		GTC CCC ATT GGC ATT CCT C
*GATA3*	107	TCA TTA AGCCCA AGC GAA GG
		GTC CCC ATT GGC ATT CCT C
*RORγt (RORC)*	111	GCA GCG CTC CAA CAT CTT CT
		ACG TAC TGAATG GCC TCG GT
*FOXP3*	63	CACCTG GCT GGG AAA ATG G
		GGA GCC CTT GTC GGA TGA
β-Actin (*ACTB*)	106	GCA TGG GTC AGA AGG ATT CCT
		TCG TCC CAG TTG GTGACG AT

bp: base pairs. Sequences obtained from the study of Lin et al. [27].

**Table 2:** Epidemiological characteristics of CKD patients with and without T2D.

Variables	Diabetic (n = 11)	Non-Diabetic (n = 11)	*p*-Value
**Gender**			
Female	3 (27.3%)	6 (54.5%)	
Male	8 (72.7%)	5 (45.5%)	
**Age (years)**	70.4 ± 8.1	64.9 ± 11.8	0.223
**ESRD Etiology**			
Glomerulonephritis	0 (0%)	3 (27.27%)	
Hypertensive nephropathy	1 (9.09%)	3 (27.27%)	
Diabetic nephropathy	11 (100%)	0 (0%)	
Chronic dysfunction kidney graft	0 (0%)	2 (18.18%)	
Polycystic hepatorenal disease	0 (0%)	1 (9.09%)	
Nephrolithiasis	0 (0%)	1 (9.09%)	
**Duration of uremia (years)**	5 ± 4	11 ± 5	**0.023**
**Duration of uremia (categories)**			
(1–3)	3 (27.3%)	1 (9.1%)	**0.038**
(4–6)	5 (45.4%)	1 (9.1%)	
(>7)	3 (27.3%)	9 (81.8%)	
**Body mass index**			
Weighted deficiency (<18.5)	1 (11.0%)	0 (0%)	
Normal (18.5–24.9)	4 (44.5%)	8 (72.7%)	
Overweight (25–29.9)	4 (44.5%)	3 (27.3%)	
**Comorbidities**			
Hypertension	8 (66.7%)	4 (33.3%)	0.099
Hepatitis C	3 (42.9%)	4 (57.1%)	0.500
Others	0 (0%)	1 (9.09%)	

Values are shown as frequency (%) or median ± interquartile range. ESRD: End Stage Renal Disease. *p*-values were obtained by Chi-square or Fisher Exact test between categorical variables and Mann–Whitney test for quantitative comparisons. *p*-values highlighted in bold indicate statistically significant differences (*p* < 0.05).

**Table 3:** Absolute cell counts of T lymphocytes in diabetic and non-diabetic CKD patients.

T Cells	Diabetic (n = 11) (Number/mm^3^)	Non-Diabetic (n = 11) (Number/mm^3^)	*p*-Value
CD4+ T lymphocyte	650.6 ± 300.4	622.8 ± 277.9	0.829
CD8+ T lymphocyte	422.8 ± 207.9	416.9 ± 126.6	0.939
CD4+ TEMRA	28.6 ± 24.2	29.7 ± 52.2	0.888
CD4+ T naïve	102.2 ± 193.4	116.5 ± 75.1	0.725
CD4+ TEM	360 ± 196.6	331.6 ± 146.2	0.714
CD4+ TCM	85.7 ± 103.4	72.5 ± 57.0	0.778
CD8+ TEMRA	185.7 ± 141.2	236.8 ± 60.9	0.439
CD8+ T naïve	49.9 ± 6.68	61.9 ± 12.5	0.401
CD8+ TEM	113.6 ± 132.2	132.9 ± 84.21	1.000
CD8+ TCM	7.6 ± 7.6	6.9 ± 5.9	0.833

TEMRA: Effector memory re-expressing CD45RA T-cells; TEM: Effector memory T-cells; TCM: Central memory T-cells. Values are shown as median ± interquartile range.

**Table 4:** Absolute cell counts and percentage of Treg cells in diabetic and non-diabetic CKD patients.

Variables	Diabetic (n = 11)	Non-Diabetic (n = 11)	*p*-Value
Treg CD4+CD25+CD127- (number/mm^3^) % of Treg CD4+ CD25+ CD127- CD4+ CD25+ (number/mm^3^) % of Treg CD4+ CD25+	14.4 ± 15.8 5.1 ± 2.6%	8.9 ± 12.03 2.8 ± 3.1%	0.091 **0.023**
	11.7 ± 13.8 4.3 ± 2.5%	6.0 ± 9.4 2.3 ± 2.4%	0.139 **0.045**

Values are shown as median ± interquartile range. *p*-values highlighted in bold indicate statistically significant differences (*p* < 0.05).

**Table 5:** Differences in genetic expression (−ΔCt) of transcription factors.

Gene	Diabetic	Non-Diabetic	*p*-Value
*GATA3*	−12.2 ± 1.08	−12.4 ± 2.63	0.72
*FOXP3*	−19.1 ± 1.90	−18.5 ± 2.16	0.86
*RORyT (RORC)*	−12.8 ± 2.40	−13.3 ± 2.19	0.45
*T-bet (Tbx21)*	−7.55 ± 1.91	−7.40 ± 1.56	0.72

Values are shown as median ± interquartile range.

## Data Availability

The raw data supporting the conclusions of this article will be made available by the authors on request.

## Supplementary Materials

The following supporting information can be downloaded at: https://www.mdpi.com/article/10.3390/biomedicines13010003/s1, Figure S1: Normalized expression of gene levels across fine PBMC subtypes. Data are shown in log_2_ of Transcripts per Million (TPM) + 1. Cell subtypes were collected as originally described in the Monaco et al., (2019) study [24].
