# Supplementary figures and images for: T-Cell Subpopulations and Differentiation Bias in Diabetic and Non-Diabetic Patients with Chronic Kidney Disease
Source: Biomedicines. 2024 Dec 24;13(1):3. doi: 10.3390/biomedicines13010003 (PMC11759818; doi:10.3390/biomedicines13010003)

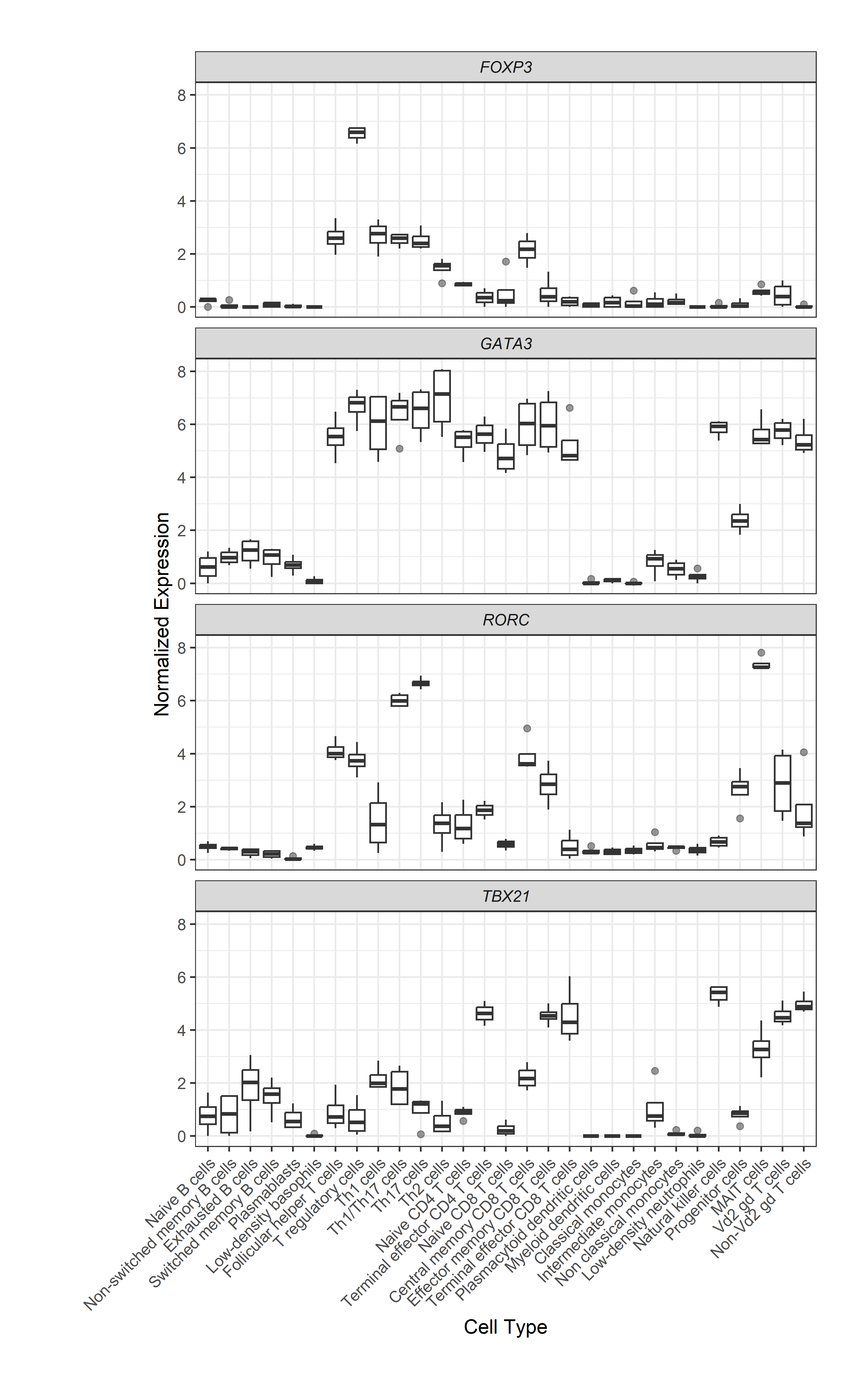

Supplement: Supplementary file 1 [file biomedicines-13-00003-s001.zip › biomedicines-3194553-supplementary.png]
